# Food Ingredients and Active Compounds against the Coronavirus Disease (COVID-19) Pandemic: A Comprehensive Review

**DOI:** 10.3390/foods9111701

**Published:** 2020-11-20

**Authors:** Charis M. Galanakis, Turki M.S. Aldawoud, Myrto Rizou, Neil J. Rowan, Salam A. Ibrahim

**Affiliations:** 1 Research & Innovation Department, Galanakis Laboratories, 73131 Chania, Greece; myrtorizou@chemlab.gr; 2 College of Science, King Saud University, Riyadh 12372, Saudi Arabia; tdawoud@ksu.edu.sa; 3 Food Waste Recovery Group, ISEKI Food Association, 1190 Vienna, Austria; 4 Bioscience Research Institute, Athlone Institute of Technology, Dublin Road, N37 F6D7 Athlone, Ireland; nrowan@ait.ie; 5 Empower Eco Sustainability Hub, Lough Boora, R35 DA50 Co. Offaly, Ireland; 6 Food and Nutritional Sciences Program, North Carolina Agricultural and Technical State University, Greensboro, NC 27401, USA

**Keywords:** SARS-CoV-2, COVID-19, vitamin D, immune system, functional foods, nutraceuticals

## Abstract

As media reports have noted, the COVID-19 pandemic has accelerated market mainstreaming of immune-boosting food bioactives, supplements, and nutraceuticals. However, most studies reporting on the potential of bioactives against COVID-19 transmission have been uploaded as preprints with little opportunity to revise content for benefit and impact. The current review discusses current best evidence and information underpinning the role of food ingredients and bioactive compounds in supporting immune functions in humans and animals, specifically in the prevention and treatment of COVID-19 disease. Up to now, some evidence from randomized population and clinical trials has suggested that vitamin D levels may be linked to COVID-19 transmission and severity. Numerous theoretical studies have pointed to polyphenols and particularly flavonoids as potential inhibitors of SARS-CoV-2 infection. There is also inconclusive evidence to support the future use of β-glucan to address COVID-19 due in part to variability in immune response arising from heterogeneity in polysaccharide branch and chain length for different sources and the absence of a standardized extraction method. To confirm the promising outcomes and hypotheses for bioactive compounds, more randomized and controlled clinical studies are needed. The results of such studies would have a profound effect on the prospects of food supplements and nutraceuticals as potential prophylaxis against COVID-19 and serve to help consumers to protect themselves during the post-lockdown recovery era.

## 1. Introduction

Over the past 20 years, much scientific focus has been trained on the identification of active ingredients in food products (e.g., vitamins) that promote human health. The terms “functional foods” and “superfoods” have become very popular concerning foods and food products that claim health benefits. To follow this trend, numerous food ingredients and active compounds have been investigated as health-promoting agents with possible antimicrobial functions, anti-inflammatory activities, and potential antiviral actions [1]. Except for the vitamins, the compounds under investigation include bioactive peptides, polysaccharides, bioactive lipids, and natural polyphenols [2]. Nutraceuticals’ demand increases despite the false efficacy claims, the concerns about quality, the lack of clinical evidence, and the self-medication implications for severe illness [3]. There is still uncertainty about the “nutraceutical” term, as these products have been reported as neither nutritious nor pharmaceutical. In many countries, there is no legal definition of “functional food” and “nutraceuticals” terms that are used interchangeably. The term “nutraceutical” (originally coined by Stephen DeFelice in 1989) is broadly used to include various naturally occurring products, such as fortified foods, functional foods, and food supplements. However, while the definition of a “food supplement” is clear, the definition of a “nutraceutical” (syncretic neologism of the words nutrient and pharmaceutical) fits in the grey area between food, food supplements, and pharmaceuticals [4]. In addition to their essential nutritional value, nutraceuticals are intended to promote health benefits, where these products typically comprise ingredients that are referred to as generally recognized as safe (GRAS) internationally. 

As the world entered the COVID-19 pandemic, the market of bioactive ingredients supporting the immune system was among the four aspects of food systems (including also food safety, food security, and sustainability) directly affected by this crisis [5,6]. In particular, consumers around the world stocked up on vitamin C and other botanical ingredients, whereas panic buying and shortages ensued [7,8]. Subsequently, the efforts of researchers and the scientific community to investigate bioactive compounds that could support the immune system, protect against viruses of the lower respiratory tract, and restrict the transmission of SARS-CoV-2 (the novel coronavirus causing COVID-19 disease) accelerated, resulting in some recent reviews summarizing the potential of natural compounds against SARS-Cov-2 and coronaviruses in general [9,10,11].

Since there is still relatively limited knowledge about SARS-CoV-2, the urgent need for fast-track prevention and treatment strategies has led researchers to publish articles in “torrents” [12]. The disadvantage of this expeditious approach to glean new knowledge is that the associated surge in recently published studies has as yet to be subjected to any review for benefit or impact. It is envisaged that incremental innovations sustaining agri-food and new products will be influenced by pressing need for new health interventions to combat COVID-19, along with responding to a surge in interest in accelerating green deal innovations and climate change [13].

Therefore, this current timely study provides a comprehensive review on the role of food and plant ingredients and active compounds against COVID-19 disease, as claimed in the torrents of early and fast-released studies. In addition, it commensurately explores the important contribution of food supplements and nutraceuticals in supporting a healthy society including considering “long COVID-19” for survivors [14]. For example, peripheral inflammation caused by COVID-19 may have long-term consequences in those that recover, leading to chronic medical conditions such as dementia and neurodegenerative disease, likely through neuroinflammatory mechanisms that can be compounded by an unhealthy diet [14]. Thus, there is a pressing need for wider access to healthy foods, and people should be made aware that healthy eating habits may reduce susceptibility to and long-term complications from COVID-19.

## 2. The Role of Food Ingredients and Active Compounds in Supporting the Human Immune System

As it is well known from mechanistic and clinical data, vitamins and folate, polysaccharides and dietary fiber, lipids, peptides, and natural polyphenols are important for the body’s immune system against viruses [1,15,16]. Table 1 presents a summary of the health benefits and possible action mode against SARS-CoV-2 virus of food ingredients and bioactive compounds. These bioactive compounds can be found in fruits and vegetables, the consumption of which is highly recommended [17,18]. For example, citrus, kiwi, and vegetables such as broccoli contain high amounts of vitamin C (ascorbic acid), which is necessary for the repair of body tissues and immune’s function [19]. Likewise, vitamin C can restrict infection of the lower respiratory tract under certain conditions [20] and prevent the common cold [21]. Vegetables such as sweet potato, spinach, and carrots contain high amounts of vitamin A (comprised of oil-soluble compounds such as β-carotene, retinol, and retinoic acid). Vitamin A is known to support immune function and protect against infections when administered at pharmacological or high dosage (10 nM or higher) due to its regulatory roles in humoral immune processes and cellular immune responses [22].

Bioactive peptides are composed of several of amino acids arranged in different configurations, and their molecular weights are <6000 kDa. Currently, more than 1500 different bioactive peptides have been reported. Although the correlation between structure and functional properties is not well established, many bioactive peptides share some structural features that include a peptide residue length between 2–20 amino acids, and the presence of hydrophobic amino acids in addition to proline, lysine, or arginine groups [23]. Bioactives are classified by their action mode (e.g., antimicrobial, antioxidant, immunomodulatory, anti-inflammatory, antithrombotic) and their binding capacity to micronutrients such as minerals. Dairy products are the primary sources of peptides, e.g., < 3 and 3–10 kDa peptide fractions obtained from fermented milks with specific *Lactobacillus plantarum* strains [24]. However, peptides can be obtained from other animal sources such as bovine blood, gelatin, meat, egg, and fish. Some plant sources of bioactive peptides are soy peanut, sorghum, pumpkins, mushroom, and rice. Peptides can also be released during the fermentation process of cheese, yogurt, and other fermented products. Peptides can be produced during cheese ripening, whereas lactic acid bacteria in general can produce a wide range of them during fermentation. For instance, some anti-inflammatory activities of peptide fractions can be obtained from fermented milk with specific *Lactobacillus plantarum* strains [24]. The probiotic strain of *Lactobacillus gasseri* SBT2055 (LG2055) has also been shown to reduce virus respiratory infection by suppressing the replication of the virus. Results demonstrated that following the respiratory syncytial infection, LG2055 enhanced the expression of IFN-β and IFN-γ at the gene level in the mice’s lungs. Another study demonstrated that several lactic acid bacteria-induced small protein molecules known as immune interferon (IFN-γ or IFN-β) in the mice’s lungs, contributing to clearance of respiratory virus [23]. Finally, another study has shown that L. paracasei strains cultured in the presence of artichokes’ phenolic extracts (4 mg/mL) have anti-inflammatory effects on dendritic cells, suggesting potential synergistic effects between different bioactive compounds [25].

Polysaccharides are complex carbohydrate polymers that are naturally found in various food sources including seaweeds and cereal grains and are also produced by microorganisms such as lactic acid bacteria. Polysaccharides have many bioactivities such as antioxidant, immunomodulatory, antidiabetic, anticancer, as well as liver and renal protective functions. A large number of studies have been conducted on the isolation and structural identification of polysaccharides from edible and medicinal mushrooms, and a total of more than forty polysaccharides were reported. In addition, these studies have shown that polysaccharides from mushrooms have antivirus activity against a wide range of viruses, including hepatitis B, influenza, enterovirus, herpes simplex virus, porcine circovirus, rotavirus, and others. For instance, *Coriolus versicolor*’s polysaccharide peptide has been demonstrated to possess immunomodulatory properties with the ability to triggers a Toll-like receptor 4 showing insignificant toxicity [26]. One explanation for this antiviral activity could be attributed to the restriction of adsorption and penetration of the virus. The polysaccharides from mushrooms exhibited potential anti-HIV activity by downregulating replication of the virus and upregulating certain antiviral chemokines (Stromal Cell Derived Factor-1 alpha, SDF-1α, and Macrophage Inflammatory Protein, MIP-1α/β). These chemokines block the coreceptors of HIV-1 in THP1 cells from leukemia patients and blood mononuclear cells [26].

Bioactive lipids comprise several endogenous molecules affecting a wide array of biological processes. Typical examples of lipids include omega-3 fatty acids and their metabolic products, carotenoids, phytosterols, and fat-soluble vitamins, phenolic lipids, and acylglycerol derivatives. Numerous studies have found an association between the consumption of certain bioactive lipids and the prevention, delay, or treatment of chronic and acute diseases such as cardiovascular disease, cancer, osteoporosis, and immune disorders. One possible protective mechanism for the bioactive lipids against virus infection is the prevention of inflammation. Bioactive lipids such as oleic acid have exhibited antiviral protection by inducing leakage and even lysis of cell membranes including the virus lipid membrane. It is also known that these bioactive lipids show phagocytic capacity of macrophages and can facilitate the removal of any damage by viral infection [27].

Polyphenolic compounds from plant foods, extracts, and food processing by-products have well known antioxidant activities [28,29,30,31,32]. Polyphenolic compounds can be subdivided into flavonoids, phenolic acid, polyphenolic amides, resveratrol, and other polyphenols. These compounds are antioxidants and also have antimicrobial and antiviral activities. Several recent studies explored the in vitro antiviral capacity of different polyphenols [33,34,35]. In addition, Vázquez-Calvo et al. [36] conducted a study related to the effect of polyphenols (e.g., epigallocatechin, epigallocatechin gallate, epicatechin, catechin, cyanidin, and delphinidin) on Dengue, Zika, and Nile viruses. These enveloped plus-strand RNA viruses are transmitted by mosquitoes, posing a serious threat to health. The results of this study showed that the aforementioned polyphenols influenced the attachment and entry step of the RNA into the host cells were dose-dependent in a 1–10 μM range and thereby reduced the infectivity of the viruses.

## 3. Food Ingredients and Active Compounds against COVID-19 Disease

The oral supplementation or intravenous administration with food bioactives and nutraceuticals has been proposed as an alternative approach against COVID-19 disease based mainly on their anti-inflammatory properties, but also to reflect their ability to inhibit viruses’ (e.g., SARS-CoV, MERS-CoV and SARS-CoV-2) activity by disrupting their protein envelopes [37]. For instance, these compounds can enhance the response of type 1 interferon to RNA viruses such as influenza and coronavirus [38]. The possible mode of action against SARS-CoV-2 of several bioactive compounds and food ingredients is presented in Table 1.

Plant secondary metabolites (e.g., β-carboline, quinoline alkaloids like cinchonine, skimmianin, dictamine, and quinine, as well as isoquinoline alkaloids such as emetine, berberine, and sanguinarine) can act as DNA intercalators (similar to chloroquine’s proposed in vitro mechanisms of action against SARS-CoV-2) inhibiting the replication of the virus [39]. Another anti-replication mechanism is through binding of angiotensin-converting enzyme 2 (ACE-2). This enzyme is a type I transmembrane metallocarboxypeptidase that mainly expressed in the renal tubular epithelium, and the vascular endothelial cells, but also in the lung, and the kidney [40,41,42]. Different studies have referred to ACE-2 as a cellular entry for SARS-CoV-2 in the body together with other factors [43,44]. In particular, the spike proteins of the virus surface binds on ACE-2 and diffuses the virus into the target cells [45]. Subsequently, compounds that exhibit a binding affinity for the core amino acid of ACE-2 have been proposed as potential preventive or therapeutic agents (these compounds mainly attack free viral particles and to a lesser extent viruses that have already entered host cells), since they are able to interfere with or avoid the host–viral interaction [39,46]. For example, omega-3 fatty acids and their metabolites inhibit angiotensin-converting enzymes (ACE, which is a precursor of ACE-2) and thus suppress ACE2-expression, reducing the availability of receptors to SARS-CoV-2 and subsequently restricting its ability to enter the target cell [37,47].

Using simulations of binding free energy and molecular dynamics, stilbene-based compounds such as resveratrol has been shown to be potential anti-COVID-19 candidates that can theoretically disrupt the virus’s spike protein [48]. Relevant action mechanisms have also been proposed for other bioactive compounds such as folic acid. Folic acid is a water-soluble form of vitamin B that can inhibit furin activity that facilitates the cleavage between ACE-2 and spike proteins of SARS-CoV-2 [49]. Those proteins have also been studied as possible targets of bioactive peptides in mitigation of SARS-CoV-2 pathology. In particular, bioactive peptides with unique amino acid sequences can mitigate such targets including, type II transmembrane serine proteases (TMPRSS2) inhibition, furin cleavage, and the renin–angiotensin–aldosterone system (RAAS) members. Based on current evidence and structure-function analysis, multiple bioactive peptides present potency to neutralize the virus [50].

Adem et al. [51] conducted a molecular docking study to identify the ability of 80 flavonoid compounds to bind 3-chymotrypsin-like protease (3CLpro), which is known to be an important enzyme for the replication of SARS-CoV. Other polyphenols and flavonoids such as protocatechuic acid, punicalagin, theaflavin gallate, kaempferol, theaflavin digallate [52], pedunculagin, tercatain, punicalin, [53], epigallocatechin gallate [54], riboflavin, daidzein, genistein, phycocyanobilin, cyanidin [55] hispidin, lepidine E [56], and hesperidin [57] have been proposed as potential inhibitors of the main SARS-CoV-2 protease in similar studies. Besides, hesperidin and other flavonoids have been referred to possess better binding poses than common drugs against COVID-19, e.g., nelfinavir, chloroquine, and hydroxychloroquine sulfate [51,58]. Another phenolic compound, quercetin, is known to display a broad range of antiviral properties that can interfere at multiple steps of pathogen virulence (virus entry, replication, and protein assembly). These therapeutic effects can be augmented by the co-administration of vitamin C. Furthermore, quercetin has recently been identified as an inhibitor of SARS-CoV-2 3CLpro [59], and also, it has been shown to act synergistically with vitamin D and have anti-viral action against SARS-CoV-2 with the proposed dosage for both substances being up to 500 mg taken two times a day for prophylaxis and mild cases [60]. Considering the different flavonoids, a thorough bioinformatic analysis of small molecules interacting with ACE-2 using the SUMMIT supercomputer indicated the structural analogue eriodictyol (5,7,30,40-tetrahydroxyflavanone) of luteolin as the most potential inhibitor of SARS-CoV-2 [61]. Finally, both quercetin and other flavonoids from different plants (e.g., litchi seeds) have shown similar activity when applied in traditional Chinese medicine [62]. In general, supplementation with dietary flavonoids up to 1–2 g/day is considered to be safe, but higher cumulative dosages should be avoided, as they can negatively affect the metabolism of the liver. Flavonoids can be obtained from different plant sources, but it is peanut shells that may affect persons allergic to peanuts, or fava beans, consumption of which could cause hemolytic anemia to Mediterranean extraction persons who lack the enzyme glucose-6-phosphate dehydrogenase (G6PD). Such patients should also not be administered the antimalarial drugs chloroquine and hydroxychloroquine, which have been advocated based on anecdotal reports for the treatment of COVID-19 [63,64].

Nevertheless, although these theoretical approaches and hypotheses about the potential role of plant secondary metabolites against COVID-19 seem to be reasonable, most of them have not been justified yet either with in vitro studies or with clinical trials. In one of the first relevant studies, Murphy et al. [65] used an in vitro lung injury model to study the activity of β-glucans from the *Shittake* mushroom. The authors using 1, 5–10 mg/mL of β-glucans reported that they reduce inflammatory responses associated with acute respiratory distress syndrome (ARDS) in vitro that includes reduced cytokine production, oxidative stress, necrosis, and apoptosis, suggesting potential for amelioration of SARS-COV-2 through a role in preventing cytokine storm. The latest occurs when white blood cells accumulate by infections such as SARS-CoV-2, releasing inflammatory cytokines. Differential in vitro immunomodulatory and pulmonary cytoprotective effects were attributed in part to variance in β-glucan structure that will have cross-cutting relevance to COVID-19 interventions. ARDS was evident in severe complications experienced by COVID-19 patients in China admitted to intensive care units [66], where the specific involvement in reducing cytokines IL-1β and IL-6 production is a strategy for COVID-19 intervention [67]. Although not studied yet, β-glucan from other sources could result in similar responses, e.g., β-glucan derived from algae (in a diet of 108 mg/kg) has been shown to enhance immune responses of weaned pigs experimentally infected with a pathogenic E. coli. In particular, the supplementation of β-glucan also reduced white blood cells, neutrophils, serum tumor necrosis factor (TNF)-α, cortisol, and haptoglobin and down-regulated (*p*  <  0.05) the mRNA expression of several immune genes (IL1B, IL6, and TNFA) in ileal mucosa of E. coli challenged pigs, compared with the control diet [68].

On the other hand, Hetland et al. [69] recently highlighted the potential for medicinal mushrooms, such as *Basidiomyceta Agaricus blazei Murill*, *Hericium erinaceus*, and *Grifola frondosa,* for prophylactic and therapeutic potential against severe lung inflammation that often follows COVID-19 infection. An AbM-based mushroom extract (Andosan™), also containing *Hericium erinaceus* and *Grifola frondosa*, has been shown to significantly reduce bacteremia and increase survival in mice with pneumococcal sepsis and to improve symptoms and quality of life of patients with inflammatory bowel disease via an anti-inflammatory effect. Hence, such mushroom extracts could have prophylactic or therapeutic effect against the pneumonic superinfection and severe lung inflammation that often complicates COVID-19 infection.

Besides, several authors highlighted the potential benefits of boosting immune response and wellbeing of individuals undergoing self-quarantine for prevention and treatment of COVID-19; such as, the consumption of foods rich in vitamins A and D, zinc, and selenium that are found in mushroom, oily fish (salmon, sardines), egg yolk, milk, cheese, whole grain and wheat bran cereals, and dark green leafy vegetables [70,71,72].

Novel therapies are urgently required to address ARDS that is associated with severe respiratory failure with approximately 40% mortality [73]. Up-regulation of pro-inflammatory cytokines associated with lung parenchyma injury is an important causation element of ARDS [74,75]. β-Glucans from Shiitake also reduced populations of a multiple-antibiotic resistant isolate *Klebsiella pneumoniae* in an in vivo lung infection model [76,77]. Findings showed that β-glucan improved lung physiological parameters, reduced white cell count protein inflammation to the lung, reduced bacterial levels in the bronchoalveolar lavage and arterial blood parameter, and supported vital partial pressure of oxygen (pO2) along with promoting lung cellular repair. The same research group recently reported on a novel means of delivering nebulized bioactives in aerosols for effective lung delivery that has potential implications for use of smart nutraceuticals in future treatment and recovery of COVID-19 patients [78]. The aforementioned also supports the findings of Bediril et al. [79], whereas in an experimental sepsis model, β-glucan attenuated inflammatory cytokine release (tumor necrosis factor-α, interleukin-1β and interleukin-β) and prevented acute lung injury. While there is emergence of smart infection models, such as for lung [65,76,77] or gut delivery [80], there is a pressing need to pursue controlled clinical studies on use of β-glucan to prove efficacy for human health.

Moreover, Luo et al. [81] reported on the use of bilberry (*Vaccinium myrtillus* Linnaeus), which investigated the anti-inflammatory effects of bilberry extract (containing 42% anthocyanin) on alleviating liver injury and croton oil-induced ear edema using a rodent infection model. In particular, bilberry extract inhibited effectively liver inflammation and croton oil-induced ear edema caused by *P. acnes*. The administration of bilberry extract suppressed the protein levels of nuclear factor-kB, tumor necrosis factor-α, and inducible nitric oxide synthase, and the increase in liver mRNA levels of interleukin-6, tumor necrosis factor-α (TNF-α), interleukin-1β, and inducible nitric oxide synthase. Bilberry extract treatment also reduced liver malondialdehyde and nitric oxide contents. These results suggest anti-inflammatory potential for future use in natural products and healthy food, such as for COVID-19 needs. Garden blue blueberry (*Vaccinium ashei Reade*) anthocyanin extracts were also recently reported for their capacity and underlying mechanisms in protecting from lipopolysaccharide (LPS)-stimulated inflammation in vitro [82]. Enzyme-linked immunosorbent assay (ELISA) studies indicated that blueberry extract restricted importantly the production of interleukin-1β, prostaglandin E2, interferon-γ, and nitric oxide. Further analysis with real-time PCR showed that in LPS-stimulated RAW 264.7 cells, the mRNA expression levels of cyclooxygenase 2, tumor necrosis factor-α, monocyte chemoattractant protein-1, interleukin-6, and interleukin-1β were suppressed. However, detailed mechanistic information underpinning the use of anthocyanin in disease mitigation remains to be elucidated.

Other reviews and studies discuss the role of vitamins (particularly A, E, and D) against COVID-19 transmission. For example, isotretinoin, which is a derivative of vitamin A, was recently suggested to restrict the activity of angiotensin-converting enzyme 2 (ACE2) [83]. In contrast, it is known that the decrease in levels of vitamin D and E in cattle could increase the infection possibilities by bovine coronavirus [84]. Besides, the increased risk of acute respiratory distress and lung injury syndrome is attributed to vitamin D deficiency. In particular, it is known that vitamin D promotes innate immunity processes, e.g., it suppresses adaptive immunity by decreasing the maturation of dendritic cells, diminishing the ability of dendritic cells to present antigen to CD4 cells, suppressing the proliferation of CD4 cells, and their differentiation into Th1 and Th17, and via promotion of Th2 and Treg [85]. Taking into account that vitamin D possesses immunomodulatory and anti-inflammatory properties as well as antiviral effects indicated by numerous studies with well-established data [86], the respective supplementation of patients may support their immune system against COVID-19 [87]. Ilie et al. [88] confirmed this using randomized population studies. More specifically, the mean levels of vitamin D for 20 European countries and morbidity and mortality caused by COVID-19 were acquired. Negative correlations between mean levels of vitamin D (average 56 mmol/L ± 10.61) in each country and the number of COVID-19 cases/1 M population (mean 295.95 ± 298.7, and mortality/1 M population (mean 5.96 ± 15.13) were observed. Vitamin D levels are severely low in the aging population especially in Spain, Italy, and Switzerland. In another effort, Daneshkhah et al. [89] combined data from two clinical studies to suggest that vitamin D may reduce COVID-19 severity by suppressing the cytokine storm in patients. A possible link between high C-reactive protein (CRP) and vitamin D deficiency and calculated an odds ratio of 1.8 among the elderly (age greater than or equal to 60 years) in low-income families and an odds ratio of 1.9 among the elderly (age greater than or equal to 60 years) in high-income families. COVID-19 patient-level data show a notable odds ratio of 3.4 for high CRP in severe COVID-19 patients. Lau et al. [90] conducted a retrospective observational study, reviewing the medical records of COVID-19 and suggesting that vitamin D insufficiency may play a role in the progress of COVID-19 disease. However, in order to validate this kind of hypotheses, more randomized controlled studies are needed, since the latest investigation did not adequately address risk-stratify subjects.

Another prevention strategy against the transmission of SARS-CoV-2 may be the consumption of herbs. Herbal medicines (e.g., traditional Chinese or Unani) are rich in dietary antioxidants such as polyphenols and vitamins and have shown some promising results for the treatment of relevant diseases. For example, *Astragulus membranaceus* extracts have shown in vitro anti-influenza virus activity [91,92]. Moreover, the consumption of ginseng root has been proposed for the prevention of influenza [93]. Hui et al. [94] revised the outcomes of population, cohort, and clinical studies that used Chinese herbal formulas for the prevention of SARS and H1N1 influenza transmission in older people and high-risk populations, suggesting the potential protective role of such formulas against COVID-19 disease. The most frequently used herbs in these studies included *Fructus forsythia* (Lianqiao), *Radix glycyrrhizae* (Gancao), *Radix saposhnikoviae* (Fangfeng), *Radix astragali* (Huangqi), *Lonicerae Japonicae Flos* (Jinyinhua), and *Rhizoma Atractylodis Macrocephalae* (Baizhu). Luo et al. [95] conducted an empirical study based on the treatment of 54 COVID-19 patients from Wuhan (China), noting that traditional Chinese medicine improved patients’ recovery. Ang et al. [96] analyzed 28 (26 Chinese and two Korean government-issued) guidelines and numerous herbal formulas, identifying different patterns for mild, moderate, severe, and recovery stages of COVID-19 disease. Glycyrrhizae Radix et Rhizoma was the most used herb in the Chinese guidelines. Silveira et al. [97] studied the possible positive effects of 39 herbal medicines on COVID-19 patients and concluded that five of them (*Althaea officinalis, Commiphora molmol, Glycyrrhiza glabra, Hedera helix* and *Sambucus nigra*) can be used as immune system boosters in early and mild cases, whereas the benefits/risks assessment of 12 (*Allium sativum*, *Andrographis paniculata*, *Echinacea angustifolia, Echinacea purpurea, Eucalyptus globules* essential oil, *Justicia pectoralis, Magnolia officinalis*, *Mikania glomerata*, *Pelargonium sidoides*, *Pimpinella anisum*, *Salix* sp, *Zingiber officinale*) others were promising. Similarly, ibuprofen showed promising results, although there was not enough evidence to endorse the use of paracetamol and/or codeine. In any case, it is important to confirm the potential protective role of the above formulas with prospective and rigorous clinical studies [94,96].

## 4. The Prospects of Food Supplements and Nutraceuticals in the Era of the COVID-19 Pandemic

The pandemic has disrupted citizen purchase behavior in a way that is referred to as the “ripple effect”; an upstream propagation of the disruptions to all actors involved in the supply chains. Subsequently, a considerable effort is required by authorities to support the primary sector in terms of economy planification, digitalization, and products’ eco-labeling [98]. To this line, forthcoming technologies for the agri-food sector will be influenced by the growing demand to produce safer and nutritious foods to meet growing populations that reflects dynamic changes in eating habits such as personalized nutrition, alternative protein sources, and attitudes towards climate change and digitization [99]. The interplay of food insecurity, malnutrition, and obesity on dietary behaviors amid the COVID-19 pandemic indicate opportunities for stakeholders to address social and structural determinants of healthy eating as a treatment strategy to improve the health span of individuals [100].

Thereby, the COVID-19 pandemic has widely affected the food sector with consumers increasingly seeking sustainable, organic, and functional foods [101,102]. Immunity was among consumers’ highest priorities, but the current pandemic has forced them to re-evaluate their eating patterns and lifestyles. According to a survey of 23,000 individual consumers by FMCG Gurus [103], 72% of European shoppers are eager to change their eating behavior by turning to healthier choices following the era of COVID-19 pandemic. Consequently, products fortified with bioactive ingredients and nutraceuticals are more popular than ever [104]. These products may be developed from food processing by-products [30,105], plants, yeast, seaweeds, algae [106], fungi, or mushrooms that reduce inflammatory responses that are typically associated with cytokine storm in severe COVID-19 patients. At the same time, there is a significant interest from existing businesses and various start-ups in animal protein alternatives, bio-based fibers, and other bio-based products (e.g., biofuels to bioplastics) that fall in the concept of the circular bioeconomy. In order to be socially accepted, the latest needs to rely on residual bio-based feedstock and waste, hence reducing its dependency on crops that compete with food markets [99].

Following this trend, several companies are releasing relevant products such as chocolate balls containing β-glucan (recovered from mushrooms) that are targeting improving children’s immunity during the post-lockdown period [107]. Subsequently, the food industry claims more recognition of immune-boosting ingredients [108], as well as highlighting the emerging need for more systematic research and collaboration with academic and governmental institutions in this field [109,110]. Indeed, despite many studies suggesting the protective role of food ingredients, the legal framework for relevant health and nutritional claims is strict, especially in Europe [111]. In this regard, organizations such as the Food and Drug Administration (FDA) and the Global Organization for Eicosapentaenoic Acid and Docosahexaenoic Acid (GOED) have released warnings and informative letters for the related food industries to avoid product claims of general immunity. It is feared that such premature or untested claims could lead to further confusion with regard to food and nutrition-related prevention or treatment of COVID-19 disease [112]. Chinese authorities have gone so far as to call out the names of products officially (e.g., probiotic pulp, vegetables, and tea) and respective companies that have unproven health claims against COVID-19 [112].

COVID-19 will present both challenges and opportunities for the development of new innovative nutraceuticals and business models that may lead to techno-socioeconomic disruption in the food ecosystem throughout the supply chain and marketplace [9]. This disruption will lead to innovative products that converge disciplines such as innovation in manufacturing, services, business processes, and Internet and communication technologies (ICT). Virtual accelerator hubs for connecting micro with small and medium enterprises (SMEs) that exploit advances in ICT through immersive technologies will become more popular for informing disruptive innovation in situ and for remote end-user applications. This tendency will enable hurdling restrictions that may come with networking and training innovators or employees in meeting rooms that may persist as a barrier to innovate for post-COVID-19 disruptors [99]. Priority is to achieve international consensus on datasets to harmonize methods for reliable and repeatable processing and to inform clinical trials [10] that can be facilitated by promoting open access to findings. COVID-19 pandemic has provided an almost instantaneous void or dearth in critical information to inform consumer market preferences, beliefs, perceptions, attitudes, and barriers towards change that will meet this particular need during and post this pandemic [113]. There is a pressing need to exploit existing or to create new multi-agency enterprise hubs related to academia that will support and accelerate innovators and businesses (such as the ones in agri-food), and full-span commercialization of products and services using the nine-stage technology readiness assessment. This fact should include off-site pilot-data generation where there is an increasing trajectory towards sustainable innovation, the green agenda, and digitization [98]. Therefore, establishing convincing and compelling evidenced-based information as to the real benefits to human health from existing and new nutraceuticals will be important moving forward. Besides, there is a trend towards online purchases of nutraceuticals as well as greater focus on security and adulteration [114].

## 5. Conclusions

The promising outcomes of the above studies along with the well-documented role of food bioactive ingredients in supporting the immune system and the supplementation of consumers’ diets with vitamins, tannins, polyphenols, flavonoids, bioactive lipids, and herbs are driving current market growth trends in the food and nutraceutical sector. Moreover, these trends will most likely continue to drive the market in the post-lockdown era [4]. However, as of 10 November 2020, at the time of this writing, there has not been adequate published evidence correlating the consumption of food bioactives with direct prevention or recovery from COVID-19 disease. Some evidence (including randomized population and clinical trials) does exist regarding the role of vitamin D against COVID-19 disease. In addition, numerous theoretical studies have suggested polyphenolic compounds (mostly flavonoids) as potential inhibitors of SARS-CoV-2 transmission. The potential for using β-glucan to address COVID-19 has also been suggested by taking into account the variability in immune response arising from heterogeneity in polysaccharide branch and chain lengths of different sources. Researchers have recommended drinking plenty of water, along with consuming foods rich in minerals such as magnesium and zinc and vitamins C, D, and E, in addition to a better life style that can boost immunity to help fight infection [115]. Because these supplements can support or improve the function of the immune system, the market prospects for nutraceuticals and functional foods within the post-lockdown period remain high, as a result of the increased interest of health-conscious consumers. Businesses will thus be seeking to fill the demand for new knowledge surrounding consumer preferences, needs, and attitudes toward nutraceuticals and functional foods in order to address the challenges and opportunities created by COVID-19 disease. The aforementioned changes that are coming will also tend to inform or even accelerate innovation across the food industry ecosystem including advances in ICT and manufacturing.

## Figures and Tables

**Table 1:** Health benefits and possible action mode against SARS-CoV-2 virus of food ingredients and bioactive compounds.

Compounds	Type of Study	Health Benefits	Mode of Action against SARS-CoV-2
Bioactive peptides (e.g., <3 and 3–10 kDa peptide fractions obtained from fermented milks with specific *Lactobacillus plantarum* strains)	In vitro	Control hormone release, anti-inflammatory, anti-hemolytic, anti-mutagenic, antioxidant and antimicrobial activities [23,24,25]	Disruption of viral spike protein [26]
Polysaccharides	In vitro In vivo	Antiviral activity stimulate ROS ^1^, reduce risk factors for chronic diseases, improve metabolism and digestibility [27]	Reduction in inflammatory responses Prevention of ARDS ^2^ [28,29,30,31,32]
Vitamins (A, C, E, and D)	In vitro Clinical	Support immune function Protect against infections Prevent common cold [19,20,21,22]	Restriction of ACE2 ^3^ activity Promotion of innate immunity [33,34,35,36,37,38]
Medicinal Herbs	Clinical In vitro	Prevention of influenza viruses [33,34,39,40]	Improve COVID-19 patients recovery [41,42]
Bioactive lipids (fatty acids, phytosterols, carotenoids)	In vivo	Enhance immune response Anti-inflammatory activities Reduce risk of cardiovascular diseases [43]	Inhibition of ACE ^4^ and restriction of virus ability to enter the cells [44]
Natural polyphenols (flavonoids, phenolic acids, stilbenes, lignans)	In vitro In silico	Anti-inflammatory, antimicrobial and antioxidant activities, antiviral capacity, prevent digestion issues, reduce the risk of chronic diseases [45,46,47,48,49,50,51,52,53]	Inhibition of viral replication Disruption of viral spike protein Inhibition of SARS-CoV-2 protease [26,54,55,56,57,58,59,60,61,62,63,64,65,66,67,68,69]

^1^ ROS: reactive oxygen species; ^2^ ARDS: acute respiratory distress syndrome; ^3^ ACE2: angiotensin-converting enzyme 2; ^4^ ACE: angiotensin-converting enzyme.
